# Polythiacalixarene-Embedded Gold Nanoparticles for Visible-Light-Driven Photocatalytic CO_2_ Reduction

**DOI:** 10.1021/acsami.2c05606

**Published:** 2022-06-17

**Authors:** Tina Skorjanc, Khaja Mohaideen Kamal, Ayesha Alkhoori, Gregor Mali, Abdul Khayum Mohammed, Zouhair Asfari, Kyriaki Polychronopoulou, Blaž Likozar, Ali Trabolsi, Dinesh Shetty

**Affiliations:** † Science Division, New York University Abu Dhabi, Saadiyat Island, Abu Dhabi, United Arab Emirates; ‡ Materials Research Laboratory, University of Nova Gorica, Vipavska 11c, 5270 Ajdovscina, Slovenia; § National Institute of Chemistry, Hajdrihova 19, Ljubljana 1001, Slovenia; ∥ Department of Mechanical Engineering & Center for Catalysis and Separations (CeCaS), Khalifa University, P.O. Box 127788 Abu Dhabi, United Arab Emirates; ⊥ Department of Chemistry & Center for Catalysis and Separations (CeCaS), Khalifa University, P.O. Box 127788 Abu Dhabi, United Arab Emirates; # Laboratoire de Chimie Analytique et Sciences Séparatives, Institut Pluridisciplinaire Hubert Curien, 67087 Strasbourg Cedex, France; ∇ NYUAD Water Research Center, New York University Abu Dhabi (NYUAD), Abu Dhab, Saadiyat Island, United Arab Emirates

**Keywords:** thiacalixarene, porous polymers, nanoparticles, CO_2_ reduction, photocatalysis

## Abstract

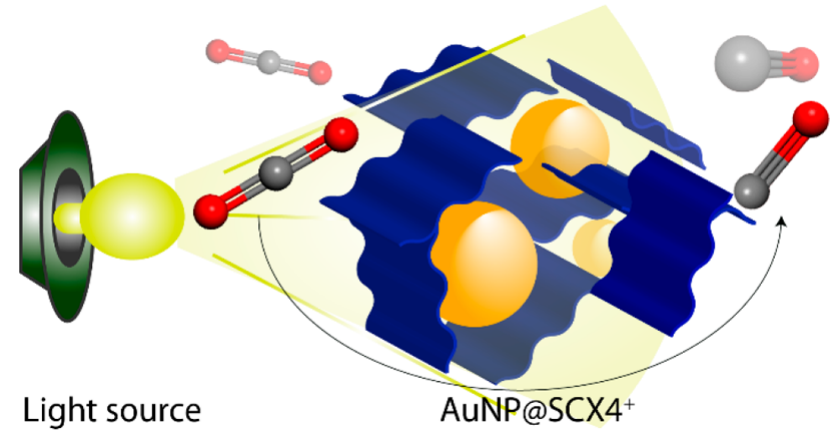

Metal nanoparticles are potent reaction catalysts, but they tend to aggregate, thereby limiting their catalytic efficiency. Their coordination with specific functional groups within a porous structure prevents their aggregation and facilitates the mass flow of catalytic starting materials and products. Herein, we use a thiacalix[4]arene-based polymer as a porous support with abundant docking sites for Au nanoparticles. The sulfur atoms bridging the phenolic subunits of thiacalix[4]arene serve as Lewis basic sites that coordinate Au atoms. Therefore, this approach takes advantage of the functional groups inherent in the monomer and avoids laborious postsynthetic modifications of the polymer. The presented system was tested for visible-light-driven photocatalytic CO_2_ reduction, where it showed adequate ability to generate 6.74 μmol g^–1^ CO over the course of 4 h, while producing small amounts of the CH_4_ product. This study aims to stimulate interest in the design and development of synthetically simpler porous polymer supports for various metal nanoparticles in catalytic and other applications.

## Introduction

With the goal of producing a stable, durable, and recyclable heterogeneous catalyst that does not self-aggregate, several classes of materials have served as solid supports for metal nanoparticles (MNPs). Unfortunately, most of these supports have serious drawbacks. In the case of activated carbons, there is the problem of leaching and aggregation of MNPs at high temperatures.^1^ Metal–organic frameworks contain labile metal–ligand bonds that limit practical applications.^2^ Zeolites have very small pores that can effectively control MNPs growth but severely limit reactant access to MNPs.^3^ In contrast, porous organic polymers (POPs) are chemically robust, have low skeletal density, and a large pore volume that facilitates mass transfer of the reactants.^4^ Most importantly, POPs have tunable structures that can incorporate highly specific chelation sites. These sites can be tuned to form strong coordination bonds with the MNPs and ultimately ensure uniform distribution of MNPs throughout the solid POP support while preventing MNPs from leaching. They can also control the growth of MNPs and influence their final size.

Several examples of chelating agents for MNPs have been reported in POPs. Salen groups were found to be effective polydentate binding sites for Pd NPs in a porphyrin-based POP.^5^ These groups contain both imine and hydroxyl functional groups that can coordinate Pd NPs. Thioether side chains introduced into one of the POP monomers before polymerization served to anchor Pt NPs in the pores of the material.^6^ Free thiol groups (−SH) obtained after disulfide bridges (−S–S−) were broken served as Au NPs coordination sites. The postsynthetic thiol–ene “click” reaction of 1,2-ethanedithiol was also reported as an effective strategy for chelating Au NPs.^7^ Importantly, most of the reported chelating and anchoring groups in POPs are introduced by laborious multistep reactions. These are often characterized by low yields, while postsynthetic modifications also present difficulties in precise characterization due to the insolubility of POPs. A strategy in which the chelating agents are present in one of the POP building blocks therefore holds immense potential.

Thiacalix[4]arene is an organic macrocycle consisting of four phenolic units linked by bridging sulfur atoms. Due to the strong affinity of thiols for various metals, monomeric thiacalixarenes have been used to anchor gold and silver nanoparticles.^8^ The macrocycle is also known for its ability to protect noble metal clusters.^9^ We thus envisioned using thiacalix[4]arene as a monomer in the synthesis of **SCX4**^**+**^, a POP that would serve as a strong support for Au NPs. In a recent review, metals on porous polymers support have been particularly praised for their high catalytic efficiency.^10^ In addition, the sulfur bridges could serve as nuclei for the growth of NPs in the **AuNPs@SCX4**^**+**^ hybrid material. Internal cavities of thiacalixarenes are also advantageous in this application, as they represent smaller pores within the larger pore system of the POP.^11^ This hierarchical structure favors the mass transport of reactants in catalysis.

Au NPs have been used for photocatalytic CO_2_ reduction in combination with various materials, including titanates,^12^ organic cages,^13^ and carbon nitrides.^14^ In most of these systems, Au NPs served as efficient absorbers of broad-range light as well as catalytic centers. Since light harvesting is the first crucial step of photocatalysis before charge transportation, and surface reactions,^15^ we were interested in exploiting these properties of Au NPs in conjunction with a porous polymer.

Herein, we have synthesized a porous thiacalix[4]arene polymer with sulfur-rich backbone via the Zincke reaction and used it to anchor Au NPs with a narrow size distribution. We investigated the well-established light absorbing properties of Au and the porosity of **SCX4**^**+**^ for the photocatalytic conversion of CO_2_ into value-added products. Irradiation with a 300 W Xe lamp produced ∼7 μmol g^–1^ CO and ∼1 μmol g^–1^ CH_4_. The material can be easily recycled and reached a total consumed electron number (TCEN) of up to 5.24, a value comparable to other reported materials.^16−18^ Thus, this study demonstrates the potential of Au NPs-porous polymer systems for photocatalytic applications.

## Materials and Methods

**SCX4**^**+**^ was synthesized by reacting 5,11,17,23-tetraamino-25,26,27,28-tetrahydroxy-thiacalix[4]arene (75 mg, 0.135 mmol) and Zincke salt (151 mg, 0.269 mmol) under microwave irradiation. A 1:1 water:EtOH mixed solvent was used and the reaction was carried out at 90 °C for 3 h. During the reaction the cream-colored reaction mixture darkened and the polymerized material precipitated. The polymer was purified with repeated water and EtOH washing, and dried in a vacuum oven at 45 °C overnight.

**AuNPs@SCX4**^**+**^ was synthesized in two steps. First, 0.0075 mmol AuCl_4_·xH_2_O and 10 mg of **SCX4**^**+**^ were vigorously stirred in 2 mL dry MeOH at room temperature. The solvent was then evaporated. Second, NaBH_4_ in MeOH (20 mg, 0.5 mmol) was added slowly. The mixture was stirred at room temperature for another 30 min, filtered, washed with methanol, and dried in a vacuum oven overnight.

### Photocatalytic Experiments

The photocatalytic activities were evaluated by reduction of CO_2_ under light irradiation. Typically, 60 mg of sample was dispersed in 8 mL of distilled water, and then the suspension was transferred into a 50 mL quartz flask. The water was dried by evaporation, and a thin film was obtained on the side of the flask. Subsequently, NaHCO_3_ (84 mg) was put into the flask, and the flask was purged with N_2_ to remove air prior to sealing with a rubber septum. Afterward, 0.25 mL of H_2_SO_4_ (2 M) was injected into the flask to react with NaHCO_3_. Finally, the sealed quartz flask was irradiated under light by using a 300 W xenon arc lamp equipped with AM1.5G filter (Newport solar simulator). The generated gas products were sampled with a syringe and analyzed using a gas chromatograph (GC, SRI-8610C) equipped with a thermal conductivity detector (TCD) and a flame ionization detector (FID) with a methanizer attachment. All the possible gas products were identified on the basis of retention time and calibrated with a standard mixture gas.

Total consumed electron number (TCEN) = (Number of reacted electrons/amount of catalyst)
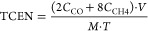
*C*_CO_ and *C*_CH4_, concentration of CO and CH_4_ (μmol L^–1^), *V*, volume of the reactor (L), *M*, photocatalyst quantity involved in the reaction (g), *T*, effective light illumination time (h).

## Results and Discussion

**Figure 1:**
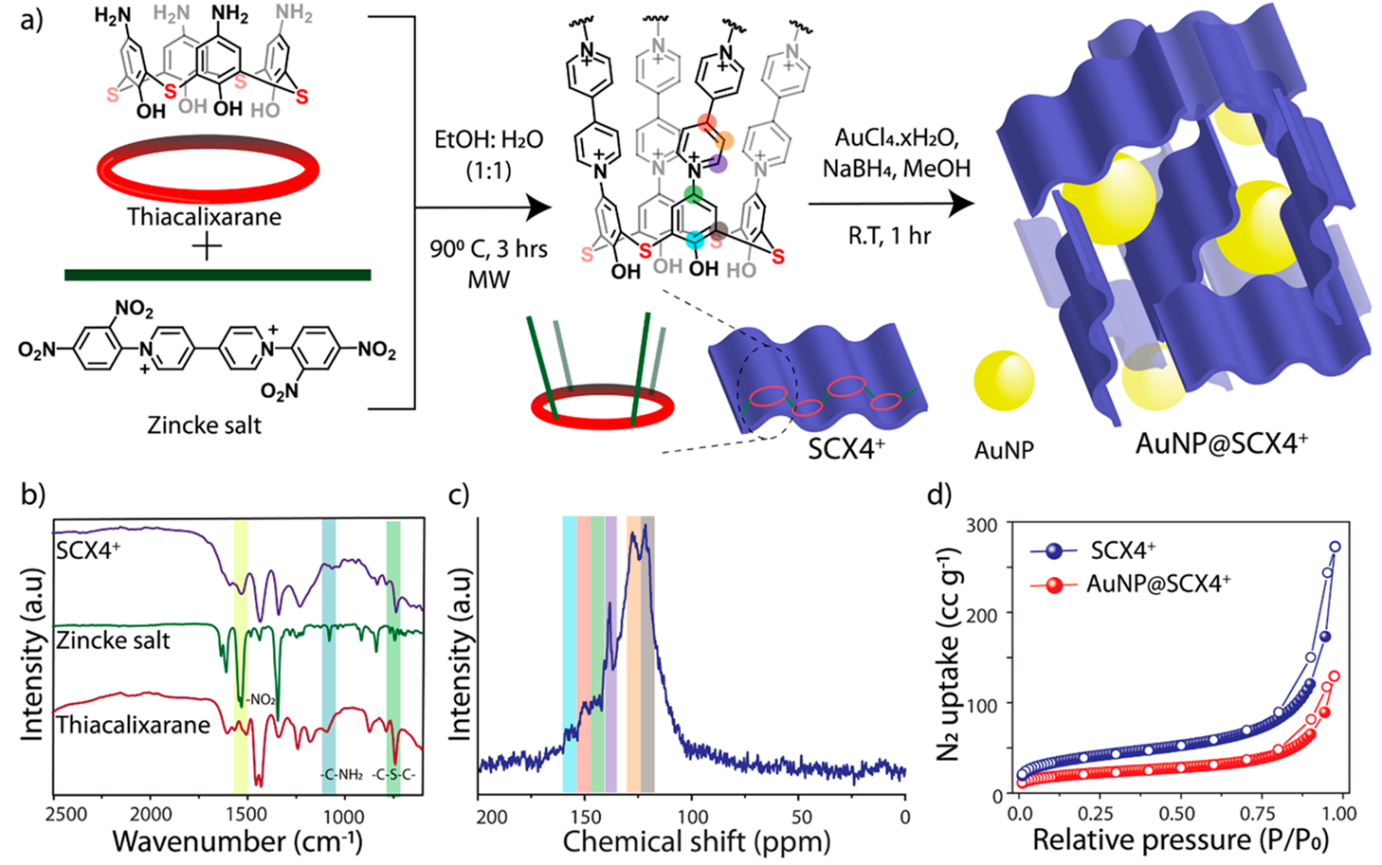
Design and characterization of the **SCX4**^**+**^ polymer. (a) Synthetic scheme for the preparation of **SCX4**^**+**^ and the **AuNPs@SCX4**^**+**^ hybrid material; (b) FT-IR spectra of the starting materials and **SCX4**^**+**^; (c) CP/MAS ^13^C NMR spectrum of **SCX4**^**+**^ with signals assigned in panel (a); (d) N_2_ adsorption isotherms for **SCX4**^**+**^ and **AuNPs@SCX4**^**+**^.

To prepare a thiacalixarene-based POP, an amino derivative of the macrocycle was first synthesized from 5,11,17,23-tetra-*tert*-butyl-25,26,27,28-tetrahydroxythiacalix[4]arene in three steps (details in the Supporting Information (SI)). The Zincke reaction was then employed to react 5,11,17,23-tetraamino-25,26,27,28-tetrahydroxythiacalix[4]arene with the Zincke salt (Figure 1a). The reaction was carried out under microwave irradiation in a 1:1 mixture of water and ethanol. The same two solvents were also used to wash the final **SCX4**^**+**^ polymer. The molecular details of the polymeric structure were confirmed by Fourier-transform infrared (FT-IR) spectroscopy (Figure 1b). We found that the signals for the −N–O bond vibrations at 1340 and 1530 cm^–1^ present in the spectrum of the linker were absent in the spectrum of **SCX4**^**+**^. Furthermore, the signals corresponding to the amine functionality in the starting thiacalixarene (−C–N stretching at 1093 cm^–1^ and – N–H bending at 1607 cm^–1^) disappeared in the final product, indicating that the amine functionality was consumed in the reaction. Meanwhile, the −C−S−C− bond vibration^19^ is present in both the starting thiacalixarene and in **SCX4**^**+**^ at ∼740 cm^–1^, as is the broad −O–H stretching peak at ∼3350 cm^–1^. These combined observations strongly suggest the formation of the polymeric structure **SCX4**^**+**^ as indicated in Figure 1a.

Cross-polarization magic-angle spinning (CP/MAS) ^13^C NMR spectroscopy was further used to confirm the structure of **SCX4**^**+**^ (Figure 1c). Due to the aromatic nature of all carbon atoms in the system, the chemical shifts occur exclusively above 120 ppm. The shielding effect of the S-bridge due to the spin–orbit heavy-atom effect on the light-atom (SO-HALA effect)^20^ positions the C atom adjacent to the bridge at 122 ppm. In contrast, the deshielding effect of the hydroxyl groups shifts the C atom adjacent to the lower rim upfield to 156 ppm. Positively charged N atoms in the bipyridinium linker are electron-deficient and therefore also cause deshielding of the neighboring C atoms. The atoms in the *para* and *ortho* positions are particularly deshielded and appear at ∼150 and 138 ppm, respectively. In addition to the FT-IR spectra, the NMR analysis confirms successful coupling of the starting thiacalix[4]arene and bipyridinium units.

The porosity of the material was evaluated by N_2_ gas adsorption measurements at 77 K. The isotherms were fitted to the Brunnauer–Emmett–Teller (BET) model to calculate the surface area of the material, which was 145 m^2^ g^–1^ (Figure 1d). **SCX4**^**+**^ is a cationic polymer with chloride counterions that can partially block the pores. In fact, some previously reported materials synthesized by the Zincke reaction were either nonporous,^21^ or only moderately porous.^22^ Therefore, the surface area of **SCX4**^**+**^ is encouraging for applications requiring interaction with small molecules such as CO_2_. The pore size distribution of the material was modeled using the nonlocal density functional theory (NLDFT). The fit resulted in a mixture of micro and mesoporosity within the material with an average pore size of 14.8 nm (SI Figure S1).

The morphology of **SCX4**^**+**^ was studied by scanning and transmission electron microscopy (SEM and TEM). SEM micrographs revealed clusters of fused structures characteristic of porous polymers (SI Figure S2). TEM images taken at higher magnification, however, showed that the clusters consisted of individual sheets. These smaller structures, with sizes in the tens of nanometers, coalesced into larger structures with micrometer sizes (SI Figure S2). Thermogravimetric analysis (TGA) indicated that **SCX4**^**+**^ exhibits enhanced thermal stability compared to its two individual components, and is generally thermally stable up to 300 °C (SI Figure S3).

**Figure 2:**
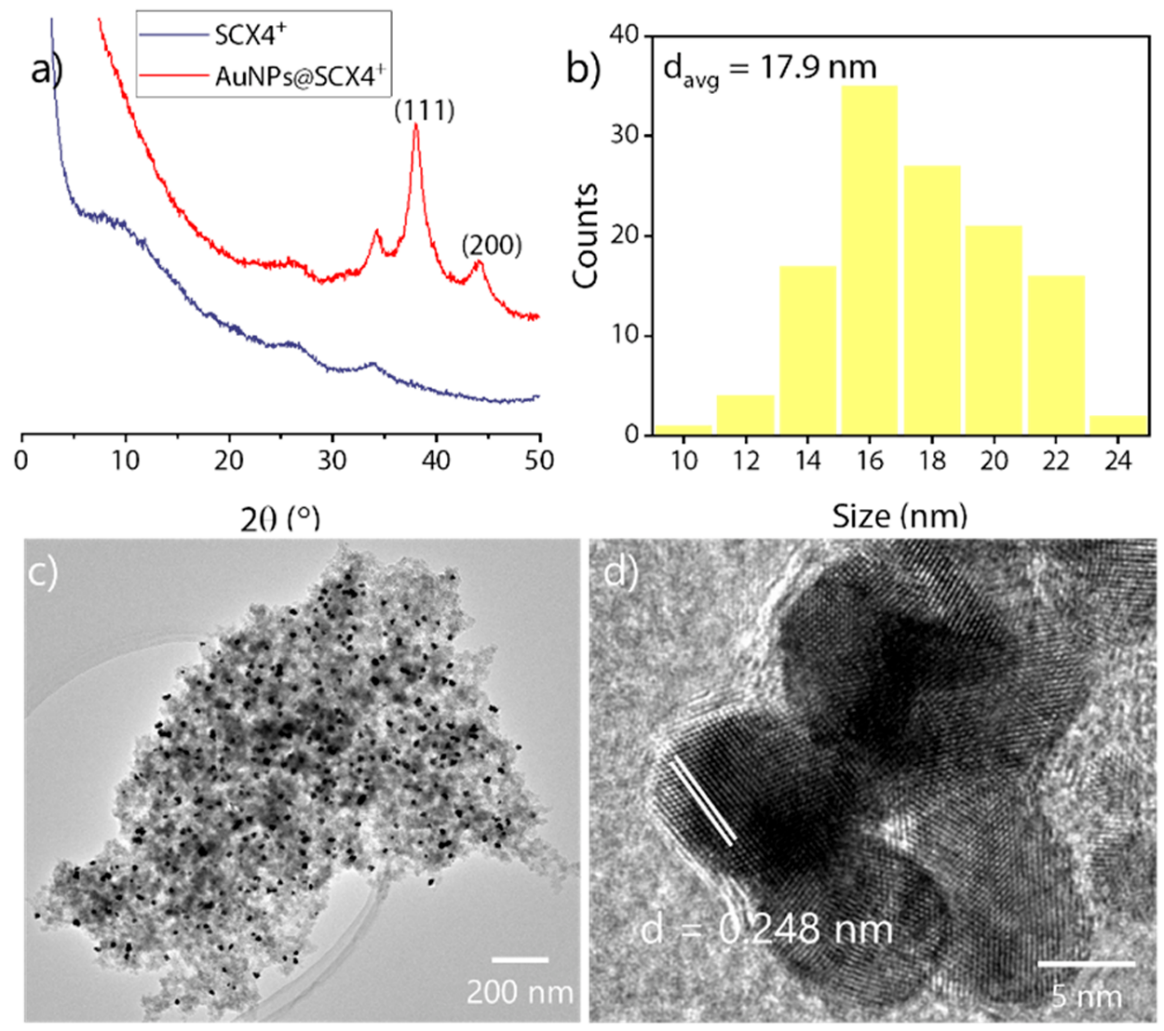
Characterization of the **AuNPs@SCX4**^**+**^ hybrid material. (a) Powder XRD patterns of amorphous **SCX4**^**+**^ and crystalline **AuNPs@SCX4**^**+**^; (b) the size distribution of Au NPs in the hybrid material; (c) a TEM micrograph showing a uniform distribution of Au NPs throughout the polymer network; (d) HR-TEM micrograph with fringes corresponding to the (111) plane of the Au NPs.

The strong chelating ability of the bridging sulfur atoms in thiacalixarene and the porosity of **SCX4**^**+**^ encouraged us to investigate the formation of noble metal nanoparticles within the polymer network (Figures 1a and 2). We chose Au NPs because of the hard and soft acids and bases (HSAB) theory that predicts the soft nature for both sulfur and gold. In addition, the interaction between Au and S is stronger than that of other noble metals.^23^ To prepare the hybrid **AuNPs@SCX4**^**+**^ material, we used Au (IV) salt in combination with NaBH_4_ as a reducing agent at room temperature (details in the Materials and Methods section). The excess of Au salt was washed off with MeOH and the dried product was fully characterized. The FT-IR spectrum of **AuNPs@SCX4**^**+**^ showed that the peak assigned to the −C–S–C– bond vibration at ∼740 cm^–1^ had a decreased intensity compared to the original **SCX4**^**+**^ and showed a shift from 737 to 733 cm^–1^ after Au NPs growth (SI Figure S4). This suggests that the sulfur atoms are now chelated to the Au NPs. While the morphology remained unchanged under SEM (SI Figure S5), several other changes were observed following the growth of the Au NPs on the POP. First, the surface area of the material decreased to 77 m^2^ g^–1^ which can be explained by some of the pores now being occupied with the MNPs, which is also confirmed by the pore size distribution (SI Figure S1). A significant decrease in the pore volume is observed for the pores of any width. On average, the pore volume decreases from 0.41 cm^3^ g^–1^ to 0.19 cm^3^ g^–1^. Second, the powder X-ray diffraction (PXRD) data clearly show that an originally amorphous **SCX4**^**+**^ contains crystalline Au NPs with peaks at 38.0° and 44.1° (Figure 2a). These signals in the PXRD pattern correspond to the (111) and (200) planes, respectively (JCPDS No. 02-1095), and are further verified by the diffraction fringes in HR-TEM measurements (Figure 2c,d). In these micrographs, the *d*-spacing was calculated to be 0.248 nm, which corresponds to the (111) plane and is consistent with the literature.^24,25^ Using the data from HR-TEM, we also calculated the size distribution of the NPs (Figure 2b). After measuring the diameters of approximately 130 NPs, the average size of Au NPs was calculated to be 17.9 nm ± 2.87 nm.

Prior to proceeding with the photocatalysis experiments, leaching tests were performed in both aqueous and methanol media. 1.0 mg of **AuNPs@SCX4**^**+**^ was incubated in 2.0 mL of solvent, briefly sonicated and the particle size distribution was measured by dynamic light scattering (DLS) (SI Figure S6). The mixture was then stirred at room temperature for 48 h. Finally, the DLS experiment was repeated and the two size distributions were compared. We note that 48 h of incubation does not lead to leaching of the Au NPs, as no structures in the range of 10–20 nm were detected. The particle size distribution in solution remains unimodal. The absence of Au NPs leaching can be attributed to the strong interaction between the Au NPs and the polymer network and is highly advantageous for photocatalysis.

**Figure 3:**
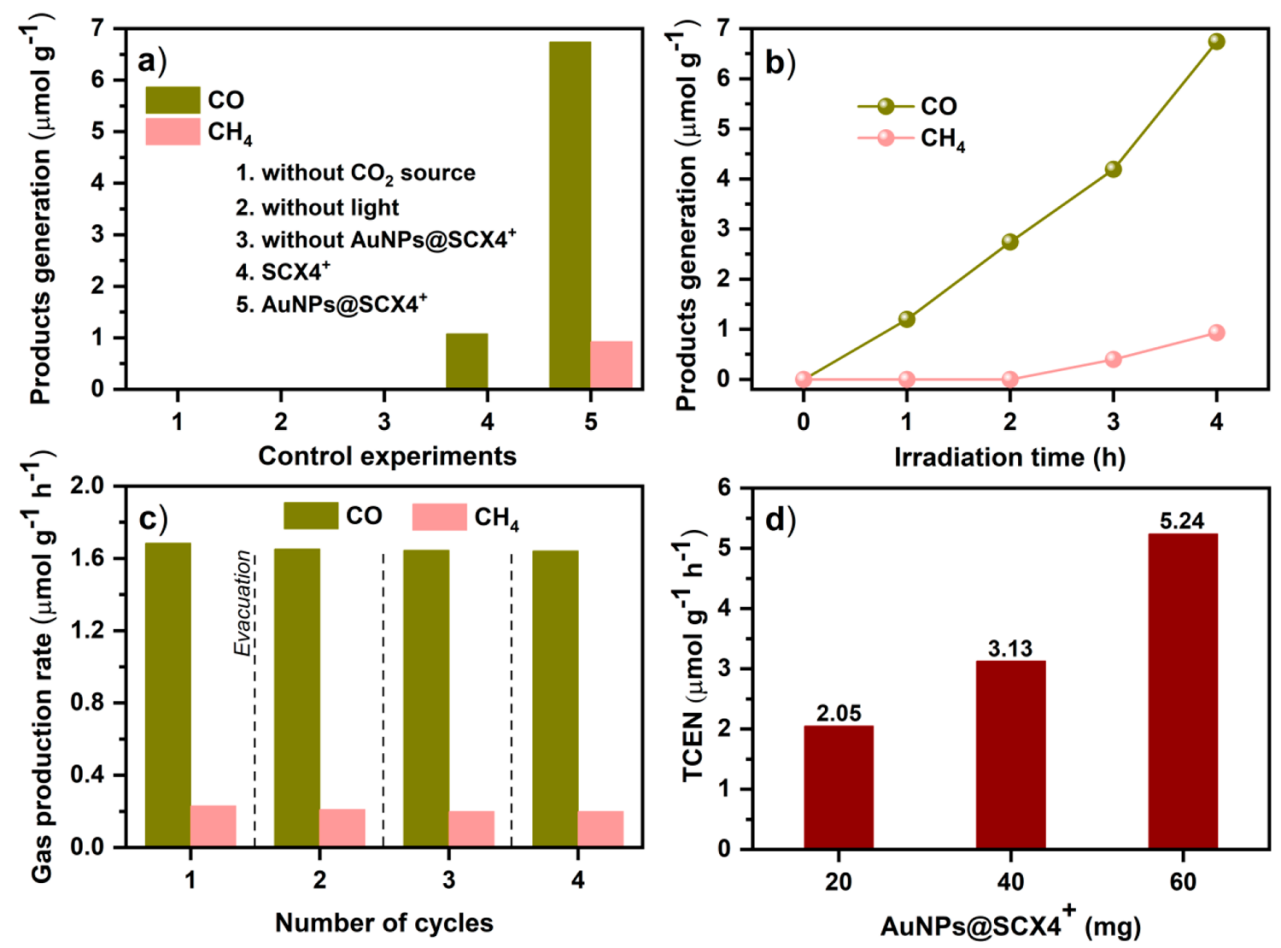
Photocatalytic performance. (a) Visible-light-driven CO_2_ reduction under various reaction conditions; (b) generation of CO and CH_4_ by AuNPs@SCX4^**+**^ as a function of reaction time; c) recycling tests of AuNPs@SCX4^**+**^ on evolution of CO and CH_4_ by photocatalytic CO_2_ reduction (each cycle 4 h); (d) total consumed electron number (TCEN) as functions of photocatalyst quantity.

After synthesizing and fully characterizing the **AuNPs@SCX4**^**+**^ hybrid material, we tested the system for photocatalytic CO_2_ reduction reactions. The photocatalytic experiments were conducted in a batch mode (SI Figure S7). As detailed in the Materials and Methods section, the material was dispersed in water, and upon water evaporation a thin film formed on the walls of a quartz flask. The CO_2_ reduction experiments were then performed in a sealed quartz flask under 300 W Xe lamp irradiation.^26^ A series of control experiments were performed to confirm that the gaseous product was produced by the photocatalytic CO_2_ reduction reaction and not by the organic decomposition of the photocatalysts themselves. These are summarized in Figure 3a. Control experiments were conducted under the following conditions: (1) in the absence of CO_2_ source (using He instead of CO_2_) (2) without light irradiation, and (3) in the absence of the photocatalyst. In all cases, no significant products were detected. This indicates that the gaseous products were generated by the photocatalytic reaction and that the presence of photocatalysts, reactant feed and light irradiation were important factors for the photoreduction of CO_2_. Among the catalysts, **SCX4**^**+**^ exhibited the lowest photocatalytic efficiency in the photoreduction of CO_2_ due to their limited photoreaction under light irradiation. This indicates that the Au NPs observed in electron microscopy imaging and PXRD measurements are crucial to photocatalytic performance.

Figure 3b shows the time courses of photocatalytic activity for CO and CH_4_ production over **AuNPs@SCX4**^**+**^ catalyst. It was proven that only gaseous products were generated that can be detected by GC. The amount of products generation increased linearly with time during the photocatalytic reactions. Quantitatively, the production rate of CO and CH_4_ over **AuNPs@SCX4**^**+**^ are reaching 6.74 and 0.90 μmol g^–1^, respectively, over the course of 4 h. When assessing the reusability of **AuNPs@SCX4**^**+**^, no significant decrease in the amounts of gaseous product was observed within four runs (each run 4 h, Figure 3c). This was in agreement with the stability evaluation with DLS, as no leakage of AuNPs was observed there. Therefore, the density of the catalytic centers does not diminish during exposure to aqueous environment.

The efficiency of the reported catalyst was evaluated by TCEN, as two different reduction products are generated.^27^ This figure takes into account the reactor volume, catalyst amount and reaction time. We tested three different **AuNPs@SCX4**^**+**^ amounts (20, 40, and 60 mg) and obtained the highest TCEN value of 5.24 μmol g^–1^ h^–1^ (Figure 3d). Although **AuNPs@SCX4**^**+**^ was synthesized without extensive postsynthetic modifications to introduce MNPs docking sites, it showed catalytic activity of the same order of magnitude as more complex reported materials composed of metal centers on polymer support (SI Table S1). Various Au NPs, Au clusters, and Au-modified species have been studied as catalysts for the same reaction (SI Table S2). Due to the variation in experimental design, amounts of catalysts, light intensities, and reported performance parameters, direct comparisons with our system are difficult.^28^

The collected experimental results allow us to propose a mechanism for the catalytic reaction. The absorption spectra of **SCX4**^**+**^ measured in the solid-state show that the porous support absorbs visible light (SI Figure S8). Subsequently, the light-promoted excited electron is transferred to the Au metal center, where the catalytic reaction takes place and CO_2_ reduction products are formed (SI Figure S9). As noted by others, Au metal centers produce CO as the main product of CO_2_ reduction.^29^ Similar mechanisms have been observed in other composite materials such as hyper-cross-linked polymer-TiO_2_-graphene,^30^ and various porous polymer-Ru composites.^10^

## Conclusion

In conclusion, sulfur-rich porous thiacalixarene-based polymer was synthesized for anchoring Au NPs. Microscopic images showed a uniform distribution of Au NPs in the polymer network and an average NP size of ∼18 nm, while DLS measurements showed no leaching of MNPs. The organic–inorganic hybrid material was tested for photocatalytic CO_2_ reduction where it showed adequate generation of CO with a TCEN of 5.24 μmol g^–1^ h^–1^. While most of the POP-MNPs hybrids for photocatalytic CO_2_ reduction use Re as the metal center, Au is added to the existing repertoire in this study.

## Supporting Information Available

Monomer synthesis procedures, FTIR spectra, TGA profiles of the monomers and polymers, TEM micrographs, photocatalysis reactor setup, gas adsorption isotherms and pore size distributions, SEM micrographs, DLS data, absorbance spectra (PDF)
