# Supplementary material for: Polythiacalixarene-Embedded Gold Nanoparticles for Visible-Light-Driven Photocatalytic CO2 Reduction
Source: ACS Appl Mater Interfaces. 2022 Jun 17;14(27):30796–801. doi: 10.1021/acsami.2c05606 (PMC9284511; doi:10.1021/acsami.2c05606)
Supplement: Supplementary file 1 — am2c05606_si_001.pdf [file am2c05606_si_001.pdf]

Supporting Information

Polythiacalixarene-embedded gold nanoparticles for visible-light-driven photocatalytic CO_2 reduction

Tina Skorjanc[a,b], Khaja Mohaideen Kamal,[c] Ayesha Alkoori,[d] Gregor Mali,[c] Abdul Khayum Mohammed,[e] Zouhair Asfari,[f] Kyriaki Polychronopoulou,[d] Blaž Likozar,[c,*] Ali Trabolsi,[a,g*] and Dinesh Shetty[e,*]

[a] Science Division, New York University Abu Dhabi, Saadiyat Island, Abu Dhabi, UAE

[b] Materials Research Laboratory, University of Nova Gorica, Vipavska 11c, 5270 Ajdovscina, Slovenia

[c] National Institute of Chemistry, Hajdrihova 19, Ljubljana, Slovenia

[d] Department of Mechanical Engineering & Center for Catalysis and Separations (CeCaS), Khalifa University, P.O. Box 127788 Abu Dhabi, United Arab Emirates.

[e] Department of Chemistry & Center for Catalysis and Separations (CeCaS), Khalifa University, P.O. Box 127788 Abu Dhabi, United Arab Emirates

[f] Laboratoire de Chimie Analytique et Sciences Séparatives, Institut Pluridisciplinaire Hubert Curien, 67087 Strasbourg Cedex, France

[g] NYUAD Water Research Center, New York University Abu Dhabi (NYUAD), Saadiyat Island, United Arab Emirates

*Correspondence: blaz.likozar@ki.si, ali.trabolsi@nyu.edu, dinesh.shetty@ku.ac.ae

General. Chemicals used for synthesis were purchased from Sigma-Aldrich as used as received. FT-IR studies were carried out on Agilent 670 IR spectrometer in the attenuated total reflectance (ATR) mode. TGA experiments were performed on a TA SDT Q600 with a heating rate of 10 °C min^{-1} over a temperature range of 70–1000 °C. Powder X-ray diffraction (PXRD) measurements were carried out on Bruker D8 Advance X-ray diffractometer with Cu Kα (λ = 1.5405 Å) radiation source operating at 40 kV and 30 mA. The patterns were recorded with divergent slit of 1/16° over the 2θ range of 1–50° with step size = 0.01°. Surface area measurements were conducted on a Micromeritics 3Flex gas sorption analyzer. Samples (~30 mg) were degassed at 85 °C for 20 h and then backfilled with N_2. Adsorption isotherms were generated by incremental exposure to ultrahigh-purity nitrogen up to 1 atm in a liquid nitrogen bath, and surface parameters were determined using BET adsorption models included in the instrument software (Micromeritics ASAP 2020 V4.00). SEM images were obtained from FEI Quanta 450FEG. TEM images were obtained from a FEI-Titan 300 operating at 200 kV. Dynamic light scattering (DLS) measurements were performed on a Malvern Zetasizer NanoSeries.

Synthetic details

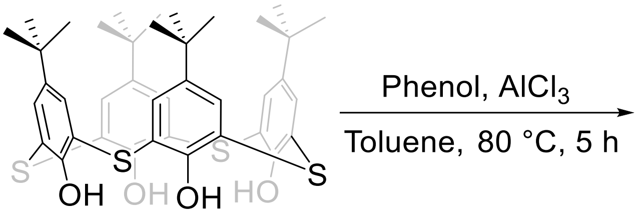

Tetrahydroxycalix[4]arene was synthesized from 5,11,17,23-tetra-*tert*-butyl-25,26,27,28-tetrahydroxythiacalix[4]arene (1.0 g, 1.39 mmol) which was dissolved in anhydrous toluene (40 mL).[1] Phenol (750 mg, 7.97 mmol) was weighed in a glovebox and added to the solution. The mixture was stirred under argon for 10 minutes at room temperature. $AlCl_3$ (2.0 g, 15.0 mmol, also weighed in a glovebox) was added slowly and the reaction mixture was refluxed at 80 °C for 5 h. After cooling down, the mixture was extracted with 3 % HCl solution (40 mL), followed by the extraction of the aqueous phase with toluene. The combined organic layers were dried over Na_2SO_4 and concentrated. Crystallization in 3 : 2 $CHCl_3$: MeOH yielded a clean product with the NMR spectrum matching the literature report.

^{1}H NMR (400 MHz, DMSO-d_6, 25 °C): 7.59 (d, 2H), 6.72 (d, 1H). ^{13}C NMR (125 MHz, DMSO-d_6, 25 °C): 157.77, 122.19, 119.26, 115.68 ppm.

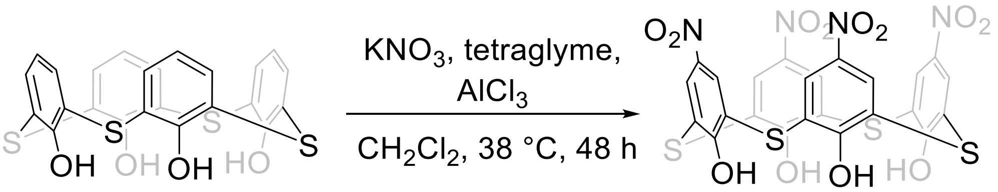

5,11,17,23-Tetranitro-25,26,27,28-tetrahydroxythiacalix[4]arene was synthesized according to a published procedure with minor modifications.[2] Tetrahydroxycalix[4]arene (600 mg, 1.20 mmol) was suspended in CH_2Cl_2 (50 mL). Tetraethyleneglycoldimethyl ether (tetraglyme; 2.0 mL, 8.97 mmol) and KNO_3 (0.25 g, 7.90 mmol) were added to the suspension and the mixture was cooled down to 0 °C. $AlCl_3$ (1.0 g, 7.50 mmol) was the added in small portions. The resulting mixture was heated under reflux at 38 °C for 48 h. After cooling down to room temperature, ~40 mL water was added slowly, and the aqueous layer was extracted with ethyl acetate three times. The combined organic layers were washed with water until neutral

pH was reached, dried over Na_2SO_4 and concentrated. The obtained product was pure based on its NMR spectrum which matched the reported one.[2]

^{1}H NMR (400 MHz, CD_3CN, 25 °C): 8.49 (s, 2H), 3.32 – 3.57 ppm (m, 22 H, tetraglyme).

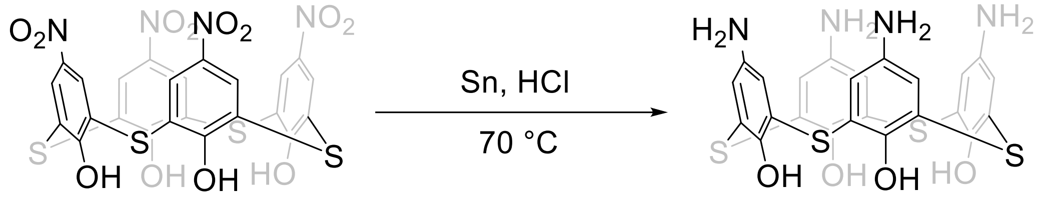

5,11,17,23-Tetraamino-25,26,27,28-tetrahydroxythiacalix[4]arene was synthesized according to a published procedure with minor modifications.[2] 5,11,17,23-Tetranitro-25,26,27,28-tetrahydroxythiacalix[4]arene (680 mg, 1.00 mmol) was suspended in concentrated HCl (40 mL). Tin was added to the suspension in small portions (3.0 g, 25.3 mmol) while stirring at room temperature. The mixture was heated at 70 °C. The reduction reaction progress was monitored by ^{1}H NMR. When the NMR signal corresponding to the starting material disappeared, the reaction mixture was cooled down in an ice bath. The white precipitate was filtered and washed with HCl, acetone and diethyl ether. The product was obtained in the pure form, and its NMR spectra resembled those reported by others.[2]

^{1}H NMR (400 MHz, DMSO-d_6, 25 °C): 7.35 ppm (s, 2H). ^{13}C NMR (125 MHz, DMSO-d_6, 25 °C): 137.32, 130.91, 122.45, 121.44 ppm.

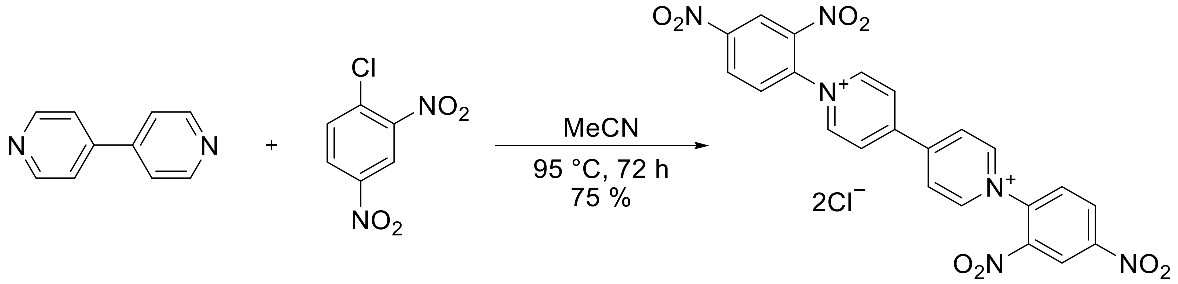

1,1'-bis(2,4-dinitrophenyl)-[4,4'-bipyridine]-1,1'-diium dichloride (tetranitroviologen, **TNV**) was synthesized by refluxing 4,4'-bipyridine (4 g, 25.60 mmol) and 1-chloro-2,4-dinitrobenzene (26 g, 89.60 mmol) in 150 mL of anhydrous acetonitrile under Ar for 72 h. After the reaction was complete, the mixture was filtered, and the solid was washed with acetonitrile (50 ml, once) and diethyl ether (40 ml, four times, followed by soaking). Pure **TNV** was obtained in 75% yield. Characterization with solution NMR afforded spectra comparable to the literature.[3]

^{1}H NMR (400 MHz, MeOD δ): δ 9.46 (d, 4H), 9.39 (d, 2H), 8.90–8.94 (m, 6H), 8.29 (d, 2H) ppm. ^{13}C NMR (126 MHz, D_2O) δ 152.66, 149.89, 146.86, 142.82, 138.24, 131.10, 130.75, 127.55, 122.82 ppm.

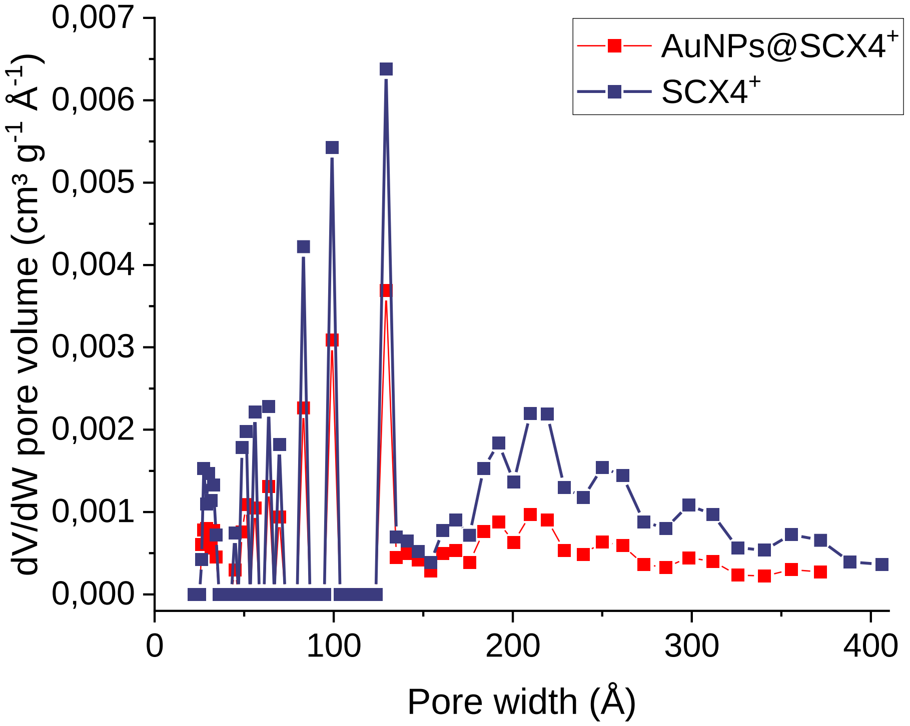

Figure S1. The NLDFT pore size distribution for **SCX4**$^+$ and **AuNPs@SCX4**$^+$.

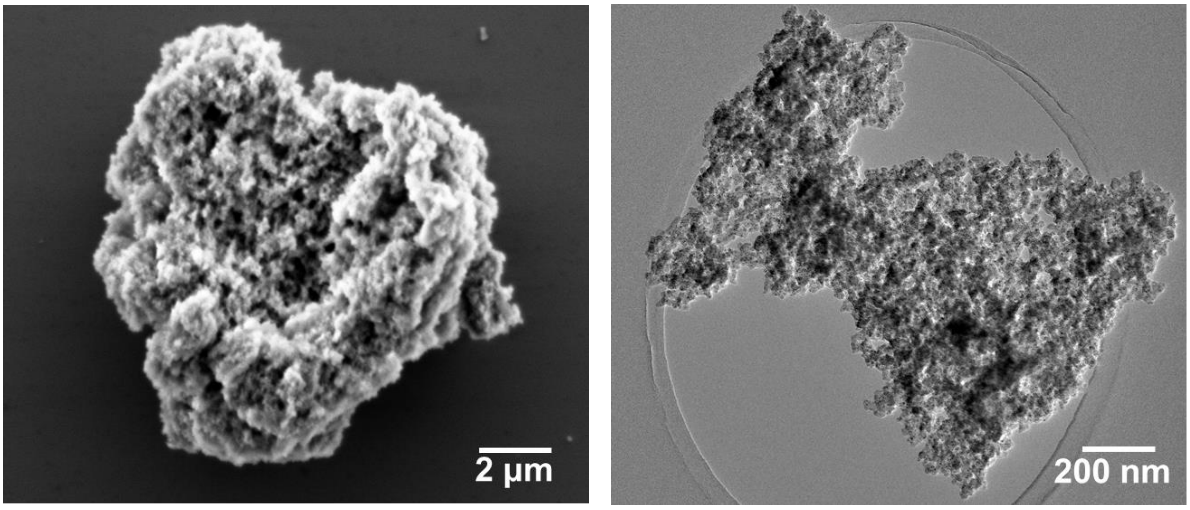

Figure S2. SEM and TEM micrographs of **SCX4**$^+$.

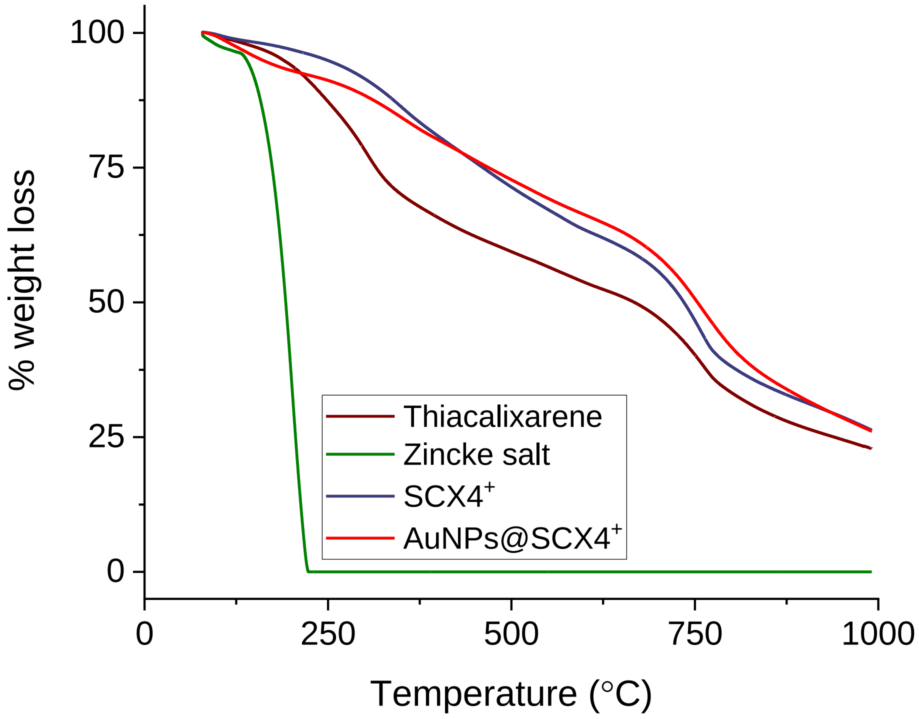

Figure S3. Thermogravimetric analysis of the starting materials, and the **SCX4⁺** polymer before and after nanoparticle deposition.

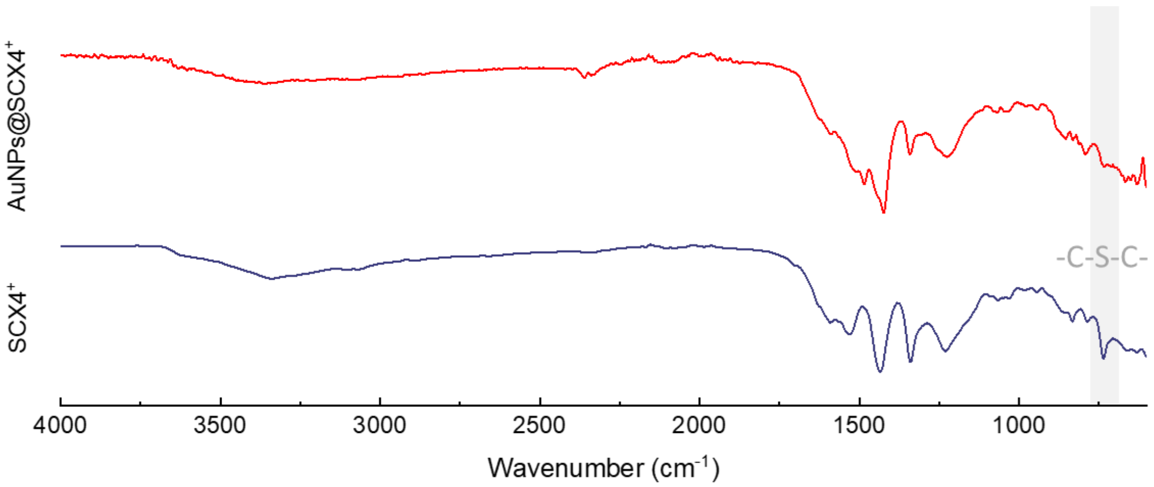

Figure S4. FT-IR spectra of the **SCX4⁺** polymer and the **AuNPs@SCX4⁺** hybrid.

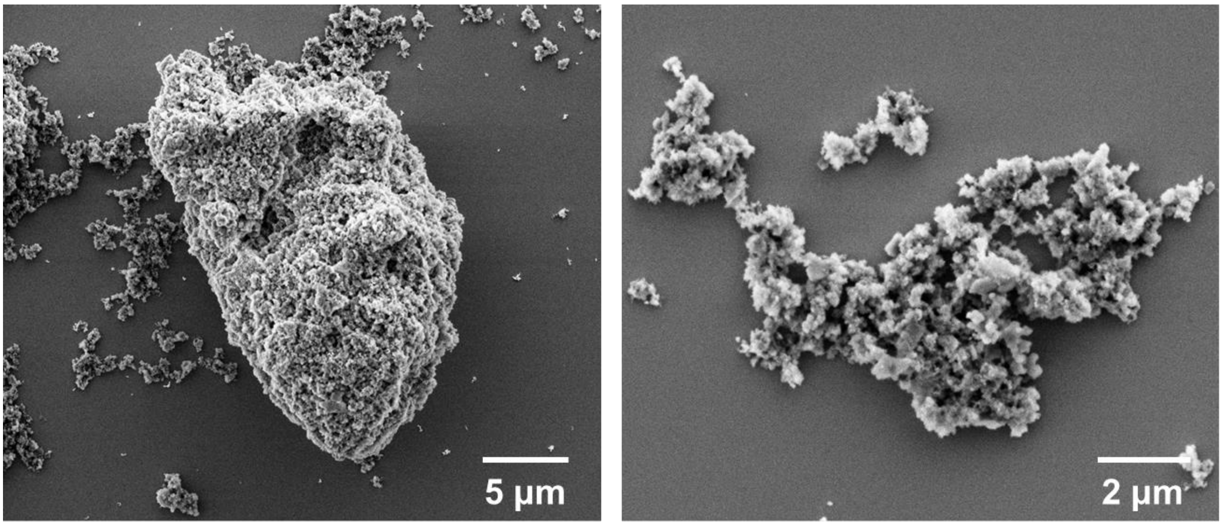

Figure S5. SEM micrographs of **AuNPs@SCX4**$^{+}$.

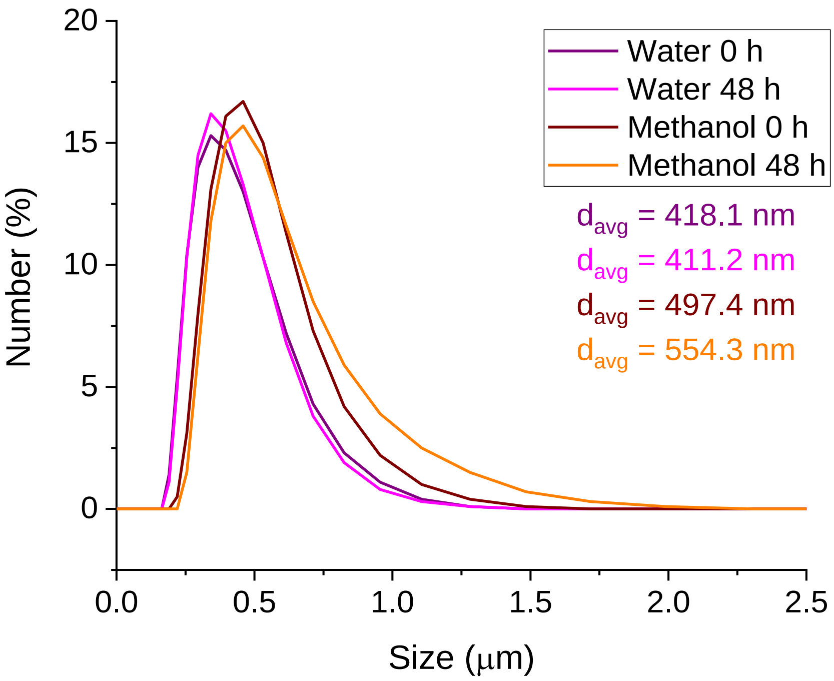

Figure S6. Au NPs leakage test from **AuNPs@SCX4**$^{+}$ in water and methanol media.

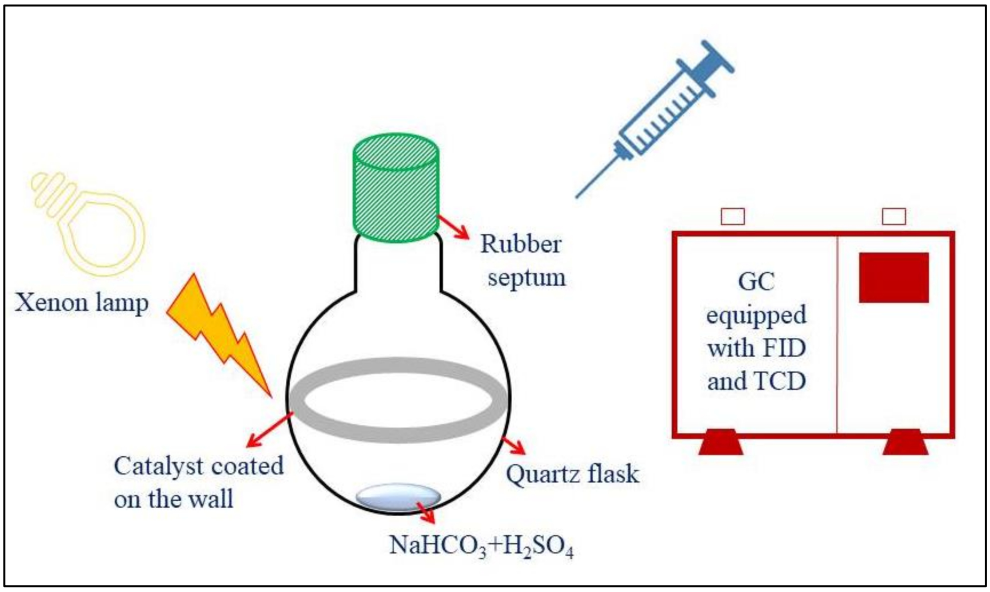

Figure S7. Schematic representation of the photocatalytic CO_2 reduction system used in this work.

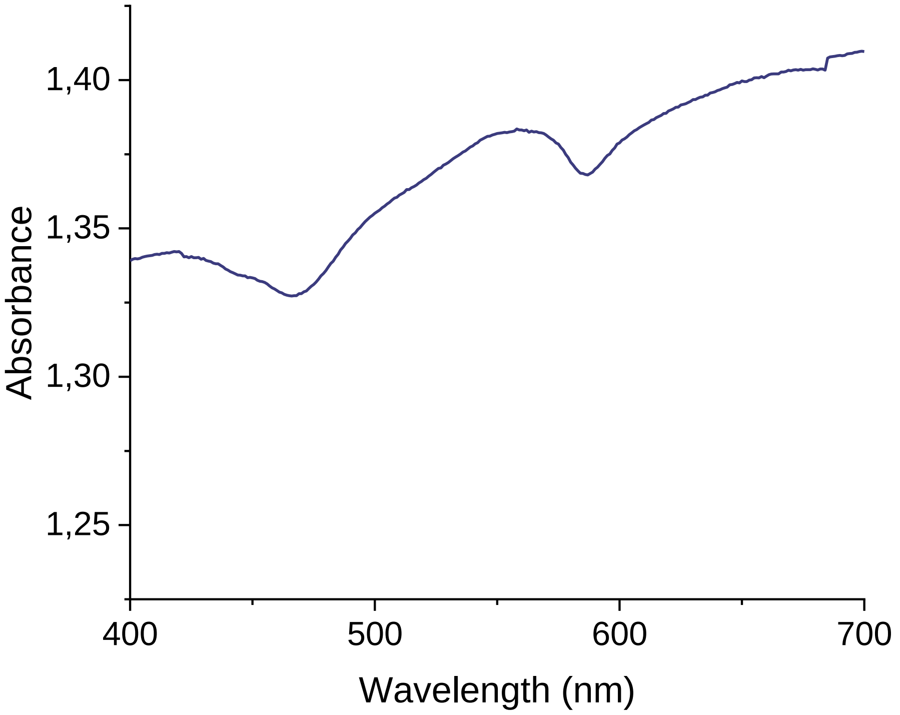

Figure S8. Solid-state absorbance spectrum of **SCX4⁺** indicates that the material absorbs visible light.

Table S1. Comparison of turnover numbers (TONs) of reported polymeric system for photocatalytic CO_2 reduction.

Material	Irradiation conditions	TON/TCEN	Reference
**AuNPs@SCX4⁺**	300 W Xe lamp	5.24	This work
**Re-COF**	225 W Xe lamp	48	[4]
**Re-Bpysp²c-COF**	300 W Xe lamp	18.7	[5]
**Re-Bpy-sp²c-COF** (Ir[dF(CF3)ppy]2(dtb py))PF_6	300 W Xe lamp	7.2	[5]
**DQTP COF-Co**	300 W Xe lamp	2.18	[6]
**1-Re**	150 W Hg lamp	5	[7]
**Re-CTF-py**	300 W Xe lamp	4.8	[8]
**Re-polythiophene**	50 W halogen lamp	20	[9]

Table S2. The performance of some Au-based catalysts for CO_2 reduction into CO.

Material	Conditions	Performance	Reference
**AuNPs@SCX4⁺**	300 W Xe lamp, 2 M H_2SO_4, 1 mmol NaHCO3	6.74 μmol g^{-1} in 4 h	This work
Au_{25} cluster	0.1 M $KHCO_3$	6.2 μmol CO h^{-1}	[10]
NHC-functionalized Au NPs	0.1 M $KHCO_3$	Faradaic efficiency = 83 %	[11]
Monodispersed AuNPs anchored onto carbon nanotube	0.5 M $NaHCO_3$	0.09 μmol CO s^{-1} cm^{-2}	[12]
Au_{25}^- cluster	0.1 M $KHCO_3$	745 L CO g^{-1} h^{-1}, TON = $6 \cdot 10^6$	[13]
Au NPs and 3% MgO-co-modified g-C3N4	300 W Xe lamp, 100 mg catalyst	423.9 μmol g^{-1} in 3 h	[14]

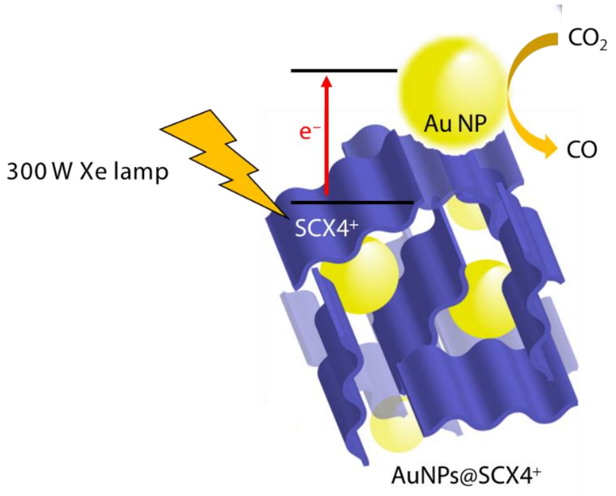

Figure S9. Schematic representation of the proposed photocatalytic reaction mechanism.

References

(1) Higuchi, Y.; Narita, M.; Niimi, T.; Ogawa, N.; Hamada, F.; Kumagai, H.; Iki, N.; Miyano, S.; Kabuto, C. Fluorescent Chemo-Sensor for Metal Cations Based on Thiacalix [4] Arenes Modified with Dansyl Moieties at the Lower Rim. *Tetrahedron* **2000**, *56* (27), 4659–4666.

(2) Desroches, C.; Parola, S.; Vocanson, F.; Perrin, M.; Lamartine, R.; Létoffé, J.-M.; Bouix, J. Nitration of Thiacalix [4] Arene Using Nitrosium Nitrate Complexes: Synthesis and Characterization of Tetranitro-, Tetraamino-, and Tetra (4-Pyridylimino) Tetrahydroxythiacalix [4] Arene. *New J. Chem.* **2002**, *26* (5), 651–655.

(3) Skorjanc, T.; Shetty, D.; Gándara, F.; Ali, L.; Raya, J.; Das, G.; Olson, M. A.; Trabolsi, A. Remarkably Efficient Removal of Toxic Bromate from Drinking Water with a Porphyrin–Viologen Covalent Organic Framework. *Chem. Sci.* **2020**, *11* (3), 845–850.

(4) Yang, S.; Hu, W.; Zhang, X.; He, P.; Pattengale, B.; Liu, C.; Cendejas, M.; Hermans, I.; Zhang, X.; Zhang, J.; Huang, J. 2D Covalent Organic Frameworks as Intrinsic Photocatalysts for Visible Light-Driven CO2 Reduction. *J. Am. Chem. Soc.* **2018**, *140* (44), 14614–14618.

(5) Fu, Z.; Wang, X.; Gardner, A. M.; Wang, X.; Chong, S. Y.; Neri, G.; Cowan, A. J.; Liu, L.; Li, X.; Vogel, A. A Stable Covalent Organic Framework for Photocatalytic Carbon Dioxide Reduction. *Chem. Sci.* **2020**, *11* (2), 543–550.

(6) Lu, M.; Li, Q.; Liu, J.; Zhang, F.-M.; Zhang, L.; Wang, J.-L.; Kang, Z.-H.; Lan, Y.-Q. Installing Earth-Abundant Metal Active Centers to Covalent Organic Frameworks for Efficient Heterogeneous Photocatalytic CO2 Reduction. *Appl. Catal. B Environ.* **2019**, *254* (October), 624–633.

(7) Liang, W.; Church, T. L.; Zheng, S.; Zhou, C.; Haynes, B. S.; D'Alessandro, D. M. Site Isolation Leads to Stable Photocatalytic Reduction of CO2 over a Rhenium-Based Catalyst. *Chem. Eur. J.* **2015**, *21* (51), 18576–18579.

(8) Xu, R.; Wang, X.-S.; Zhao, H.; Lin, H.; Huang, Y.-B.; Cao, R. Rhenium-Modified Porous Covalent Triazine Framework for Highly Efficient Photocatalytic Carbon Dioxide Reduction in a Solid–Gas System. *Catal. Sci. Technol.* **2018**, *8* (8), 2224–2230.

(9) Apaydin, D. H.; Tordin, E.; Portenkirchner, E.; Aufischer, G.; Schlager, S.; Weichselbaumer, M.; Oppelt, K.; Sariciftci, N. S. Photoelectrochemical Reduction of CO2 Using Third-Generation Conjugated Polymers. *ChemistrySelect* **2016**, *1* (6), 1156–1162.

(10) Kauffman, D. R.; Alfonso, D.; Matranga, C.; Qian, H.; Jin, R. Experimental and Computational Investigation of Au25 Clusters and CO2: A Unique Interaction and Enhanced Electrocatalytic Activity. *J. Am. Chem. Soc.* **2012**, *134* (24), 10237–10243.

(11) Cao, Z.; Kim, D.; Hong, D.; Yu, Y.; Xu, J.; Lin, S.; Wen, X.; Nichols, E. M.; Jeong, K.; Reimer, J. A.; Yang, P.; Chang, C. J. A Molecular Surface Functionalization Approach to Tuning Nanoparticle Electrocatalysts for Carbon Dioxide Reduction. *J. Am. Chem. Soc.* **2016**, *138* (26), 8120–8125.

(12) Huan, T. N.; Prakash, P.; Simon, P.; Rousse, G.; Xu, X.; Artero, V.; Gravel, E.; Doris, E.; Fontecave, M. CO2 Reduction to CO in Water: Carbon Nanotube–Gold Nanohybrid as a Selective and Efficient Electrocatalyst. *ChemSusChem* **2016**, *9* (17), 2317–2320.

(13) Kauffman, D. R.; Thakkar, J.; Siva, R.; Matranga, C.; Ohodnicki, P. R.; Zeng, C.; Jin, R. Efficient Electrochemical CO2 Conversion Powered by Renewable Energy. *ACS Appl. Mater. Interfaces* **2015**, *7* (28), 15626–15632.

(14) Li, N.; Huang, M.; Zhou, J.; Liu, M.; Jing, D. MgO and Au Nanoparticle Co-Modified g-C3N4 Photocatalysts for Enhanced Photoreduction of CO2 with H2O. *Chinese J. Catal.* **2021**, *42* (5), 781–794.
